# Sustainability Study of Concrete Blocks with Wood Chips Used in Structural Walls in Seismic Areas

**DOI:** 10.3390/ma15196659

**Published:** 2022-09-26

**Authors:** Simon Pescari, Laurentiu Budau, Razvan Ciubotaru, Valeriu Stoian

**Affiliations:** Department of Civil Engineering and Building Services, Faculty of Construction, Polytechnic University Timișoara, Victoriei Square, No. 2, 300006 Timișoara, Romania

**Keywords:** sustainable materials, wood chips, theoretical study, concrete blocks, structural elements

## Abstract

The concept of sustainability has become a priority in the construction field, in a context where there is an increasing discussion about reducing carbon dioxide emissions, as the construction industry is one of the most polluting industries with a focus on the production of building materials. At present, the classic solution used for structural masonry walls worldwide is the ceramic block. Given that the production of ceramic blocks represents an environmentally polluting process, the alternative solution of using concrete blocks with wood chips is proposed. The proposed solution is more environmentally friendly, both in terms of production technology and materials used, as it is made of wood chips, wood being a sustainable material. These types of blocks are currently used in non-seismic areas due to their poor structural performance. This paper deals with a study on the use of recyclable materials, such as wood chips, from waste materials and aims to propose viable solutions for the use of this type of blocks for structural walls in seismic areas. Two solutions, including concrete blocks with wood chips, have been proposed and numerical analyses have been carried out. Numerical analyses were also carried out for the classical solutions, so that, finally, a comparison could be made between them from a structural point of view. Following the numerical analysis of four types of walls, the two proposed solutions of concrete blocks with wood chips had the best results in terms of force–displacement relationship. Moreover, the quantitative results are presented in a force–displacement graph for the four wall types. This stage represents the first phase of the research, while phase II will continue with experimental tests of the proposed solutions.

## 1. Introduction

A lot of studies on the use of structural walls made of ceramic blocks in seismic areas are found in the literature. Studies exist about concrete blocks with different materials such as ceramic aggregates, plastic fiber, ornamental stone waste, and alkali-activated slag [1,2,3]. The topic of numerical modeling of walls made of ceramic blocks is extensively researched and debated in specialized works [4,5,6,7,8,9,10]. The weak mechanical characteristics of the walls of brick masonry that can even lead to degradation in the structure due to moderate seismic actions [11,12,13,14,15,16], makes finding a solution regarding the use of walls to be a research topic of great interest. On the other hand, the European Commission has made an intense campaign on environmental issues lately which also consisted in the elaboration of scientific reports on this subject [17,18,19]. As a result, several directives have been adopted on the reduction of the environmental impact of the construction sector. The production of building materials, the production of which significantly pollutes the air quality by releasing dust, and then the entire construction process, which is also highly polluting and unfriendly to the environment [20,21].

Sustainability is the key word around which most of the European directives adopted in the field of construction have focused lately [22,23,24]. The desire to have a healthier environment, breathe cleaner air, and mitigate the effects of global warming led to the adoption of these directives. In addition to reducing the number of pollutants emitted by the production of various building materials, the aim is, also, to reduce greenhouse gas emissions caused by the burning of fossil fuels used to heat buildings [25,26]. The manufacture of ceramic blocks used in construction is a process that generates pollution by releasing dust into the atmosphere. The environmental factors affected by the pollution generated are water, air, soil, and vegetation [4,27]. Thus, nowadays, alternative solutions are being sought for the use of building materials that meet environmental requirements.

The novelty of this study is the use of concrete blocks with wood chips in seismic areas. Finding a solution such that these sorts of blocks are practical in seismic areas can have a considerable benefit, bringing innovation in terms of building structural walls in previously mentioned areas.

Consequently, this work is to analyze concrete blocks with wood chips, a solution which is known to use wood as a raw material, as wood is considered a sustainable material, and, furthermore, to find a viable structural solution so that these blocks can be used in seismic areas. Due to the fact that these blocks have poor mechanical characteristics in terms of tensile strength, compressive strength, and modulus of elasticity, solutions must be proposed to improve the configuration of the blocks so that they become viable solutions for use in seismic zones. In addition, the paper presents a comparison between ordinary ceramic blocks and the proposed solutions for concrete blocks with wood chips.

Therefore, due to the aspects mentioned above, and due to the European directives which stipulate the need for construction with a low impact on the environment, as well as starting from the topic of evaluation of structural walls of brick masonry [28,29]. This paper addresses the topic of walls made of concrete blocks with wood chips in seismic areas that can prove to be a long-term solution for reaching structural safety, the environmental, and sustainability requirements [30]. Wood can provide added value in terms of energy performance of the building due to its thermal conductivity [31]. The first phase of the work aims to carry out numerical analyses, after which experimental tests are planned for the proposed solutions.

## 2. Concrete Blocks with Wood Chips Solutions

The high wood content masonry blocks have been known worldwide for more than 50 years. The first element of this type appeared in 1934, which is nowadays a quite widespread and frequently used product, being produced and put into use all over the world. The conformity and quality of these products are certified by procedures, which strictly comply with the legislation in force, and all items have a declaration of performance. These masonry blocks are a prefabricated building element made of a mixture of cement, water, and mineralized wood chips. Renewable resources, such as wood chips, are needed to produce the base material and can be in different forms. The wood used to make these elements is waste wood made of old pallets and cuttings that would have been stored as waste, thus releasing carbon dioxide into the atmosphere. Composition of concrete block with wood chips is detailed in Table 1.

**Figure 1:**
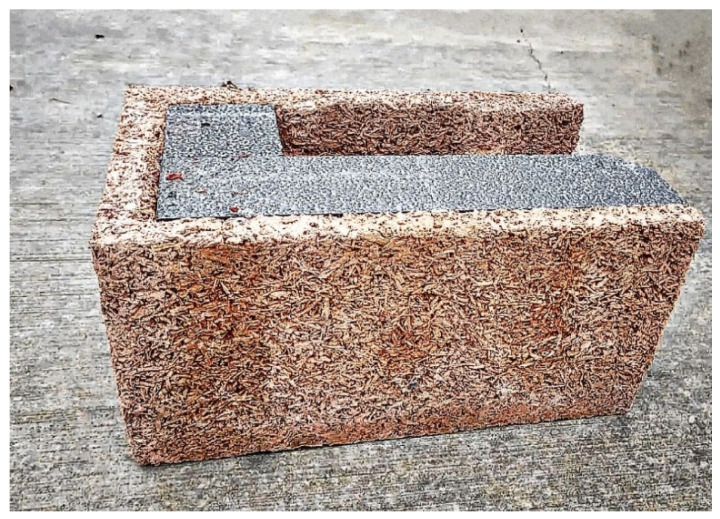
Concrete block with wood chips.

Because the wood is mineralized and enclosed in cement it will not burn or rot, so the embedded carbon is captured and permanently locked in. Concrete blocks with wood chips are fully recyclable, they contain no toxic elements and are safe for the environment as they are truly green and healthy products. These types of blocks are highlighted in the following images, thus, in Figure 1, a concrete block with wood chips of greater thickness partially filled with concrete is illustrated. The image also shows the texture of the material resulting from the mix of concrete and wood chips.

The configuration of the concrete block with wood chips consists of longitudinal walls and transverse ribs delimiting the interior voids, the blocks also act as lost formwork. These types of elements are used in the construction of all types of walls, either interior walls, end walls, partition walls, or load-bearing walls. The thickness of concrete blocks with wood chips ranges from 15 to 37.5 cm, depending on the type and purpose of the construction: houses, blocks of flats, public buildings, or industrial buildings. The assembly of the elements is performed by hand, with the blocks being connected using concrete poured into the elements.

This type of system combines all the advantages of wood and concrete, being thermally and acoustically insulating and has a very good heat storage capacity. This system gives an advantage in terms of water vapor permeability, ensuring a balance between the air temperature in the room and its relative humidity, thus ensuring a healthy and pleasant environment. The wood chip blanket gives an advantage in terms of thermal insulation properties as well as the heat storage capacity of the concrete core, all of which, in turn, give cost advantages for heating during the cold season or for cooling the air in periods of high temperatures. For a standard concrete block with wood chips, with a thickness of 375 mm and a length of 500 mm filled with thermal insulation of 175 mm and a height of 250 mm, the heat transfer resistance for an unglazed wall is R = 5.26 m^2^ K/W and the heat transfer coefficient for an exterior wall with plaster interior plaster + lime-cement exterior plaster is U = 0.18 W/m^2^ K [5].

Considering the fire resistance of these types of blocks, although they have a wood chip content of 85% and some concrete blocks with wood chips also include a polystyrene thermal insulation layer, which is due to the manufacturing process they are 100% fire resistant REI 180; a resistance higher than 180 min for a plastered wall. Regarding the load-bearing characteristics of these wood chip blocks, there is research, currently, which shows that the characteristic compressive strength is comparable to that of a low-quality ceramic block Rc, average = 2.5 MPa, modulus of elasticity E = 300 MPa, and specific gravity g = 700 kg/m^3^ [32]. Like commonly used ceramic blocks concrete blocks with wood chips have a low tensile strength. The ability to take up tensile stresses can be increased by introducing appropriate amounts of reinforcement by effective anchoring. Referring to the load bearing system, for the concrete block system with wood chips, the loads acting on them are taken up by the concrete core. The concrete core can be plain or reinforced and as a method of placing, it must be cast in place in the voids of the elements. Mix ratio of concrete use is detailed in Table 2.

## 3. Proposals for Evaluation against Existing Walls

### 3.1. Solid Brick Wall

**Figure 2:**
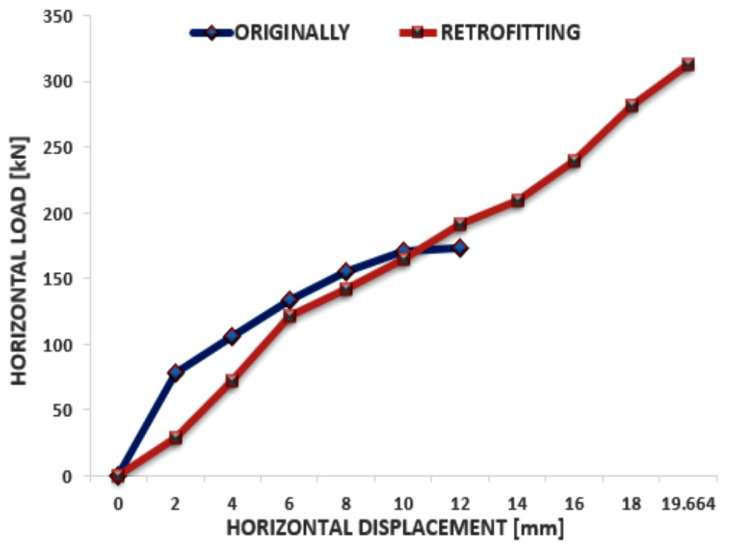
Graph load—displacement solid brick wall originally and retrofitting.

The evaluation of solid brick masonry walls has been the subject of recent research carried out in the Department of Civil Construction and Installations of the University “Politechnica” of Timisoara. The aim of the research was to evaluate the behavior of solid brick masonry walls to lateral loading both in their initial and reinforced state. The research was carried out for a wall with the following dimensions: length = 150 cm; height = 150 cm; thickness = 24 cm, with the ceramic blocks having an average compressive strength of 10 N/mm^2^ and a cement-based mortar with a compressive strength of 13–16 N/mm^2^. The tested wall was subjected, simultaneously, to a vertical force applied constantly and a horizontal force applied cyclically by means of an actuator. The results of the experimental test revealed the shortcomings of solid brick masonry walls in terms of seismic energy dissipation induced by horizontal forces. The wall failure occurred through a diagonal crack along the wall at a horizontal load of 173 kN, while the maximum horizontal displacement reached 10.488 mm. The consolidation solution achieved was the consolidation with metal fiber reinforced mortar. A high-strength, metal fiber-reinforced mortar was used for the reinforcement, which has superior characteristics in terms of tensile strength. The test results are shown in the graph in Figure 2.

The conclusions of the study show that after the wall reinforcement, the bearing capacity increases by 80% compared to the initial one, and the wall displacements also increase two-fold, thus demonstrating the increase in wall ductility and seismic energy absorption capacity. [33]

### 3.2. Masonry Wall of Ceramic Blocks with Vertical Voids

Moreover, within the Department of Civil Construction and Installations of the University “Politechnica” of Timisoara, research was carried out on masonry walls made of ceramic blocks with vertical voids, being tested a group of nine walls, consisting of both simple masonry and masonry confined with column. The elements are made of Porotherm 25 vertically hollow ceramic blocks, with dimensions 375 mm × 250 mm × 238 mm with an average compressive strength of 10 N/mm^2^ and a cement-based mortar with a compressive strength between 5–6 N/mm^2^. The failure of the elements made of simple masonry occurred at horizontal loads between 105–140 kN with horizontal displacements between 5.4–11 mm. For elements made of masonry confined with studs, an increased load-bearing capacity was observed at horizontal actions, thus failure occurred at horizontal loads between 200–230 kN with maximum horizontal displacements of 6.0 mm.

The reinforcement solution implemented was polymer composite reinforcement. The tests carried out for the reinforcement of the walls with composite materials showed a recovery of the maximum horizontal force with values between 80% and 115%, and for the maximum lateral displacements, a recovery of between 50% and 80%. The walls reinforced with polymeric materials had a lower ductility than the walls in the original condition. [34]

### 3.3. Concrete Block Wall with Wood Chips

The study of masonry walls made of concrete blocks with wood chips is proposed to be the next research topic carried out in the Department of Civil Construction and Installations of the University “Politechnica” of Timisoara. The research will focus on walls made of concrete blocks with wood chips with dimensions 150 cm × 150 cm × 25 cm. The test procedure adopted will be similar to the other two cases mentioned above, namely the application of a constant vertical force alongside a cyclic horizontal force. The purpose of the tests is to evaluate the behavior of the walls to lateral loading. It is proposed to evaluate a group of three elements for a better accuracy of the results. Compared to the classical ceramic blocks, the advantage of these blocks is the use of wood chips which are considered as sustainable materials.

## 4. Numerical Analysis

In the research, a series of four numerical models have been built to simulate the behavior of each proposed masonry type under lateral loading. The analyzed elements were modelled as shell elements. Regarding the boundary conditions of the FEM models in the numerical analysis it was considered that at the lower part of the element, the displacements and rotations of the element were blocked, simulating the embedding of the element, while at the upper part of the element the rotations were blocked as well as the vertical displacement at the same time being allowed the displacement of the element in the horizontal plane. Moreover, with reference to the loading conditions, the numerical models were subjected to general static vertical loads with values between 150–200 kN, and, also, to a dynamic load of the Static Riks type intended to simulate the effect of a horizontal movement induced by the earthquake.

The discretization of the model was carried out at the whole element level, so that the block-mortar assembly is idealized as a homogeneous medium with equivalent properties.

### 4.1. Solid Brick Masonry Wall URM

The numerical model is designed to demonstrate that the assumed experimental model is properly accepted. The numerical model is designed to demonstrate that the assumed experimental model is properly accepted in the calculation, therefore, we used an average compressive strength of 10 [N/mm^2^] for the solid brick masonry. Moreover, in the analysis we used a plastic strain of 0.0025, a failure ratio R1 = 1.16; R2 = 1.08, and tension stiffening with displacement of 1.5. The modulus of elasticity of the ceramic block was of E = 13,000 N/mm^2^ with Poisson ratio of 0.25. As such, a model has been constructed which fully reflects the study carried out in the laboratory. Thus, a vertical force V = 200 kN and a horizontal increase force was applied in order to observe the mode and moment of failure of the element. The analysis was performed using the ABAQUS program using the finite element method. Following the numerical simulations performed, the maximum horizontal force recorded was H = 144 Kn with a maximum displacement at the top of the element of 10.64 mm.

**Figure 3:**
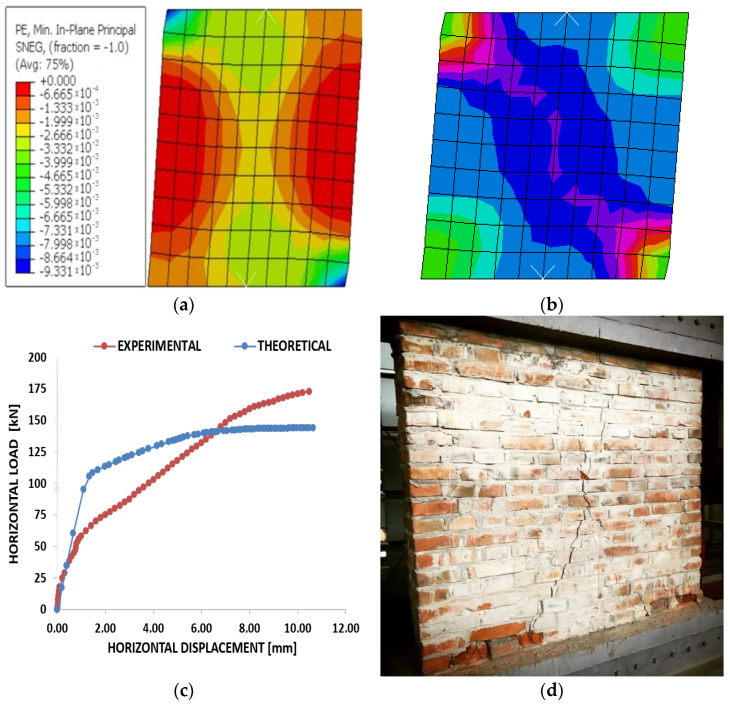
Compression plastic strain (**a**); strain components at integration point (**b**); graph of force–displacement to URM experimental and theoretical (**c**); failure mode of wall of solid brick masonry from the experimental program (**d**).

In the following pictures are illustrated the deformed element with the displacement at the top of the element (Figure 3a), the deformation mode as well as the area where the failure of the element occurred (Figure 3b), the force–displacement graph for the numerical and experimental analysis performed (Figure 3c), and the failure mode of wall of solid brick masonry from the experimental program (Figure 3d).

### 4.2. Masonry Wall from Ceramic Blocks with Vertical Holes VHB

**Figure 4:**
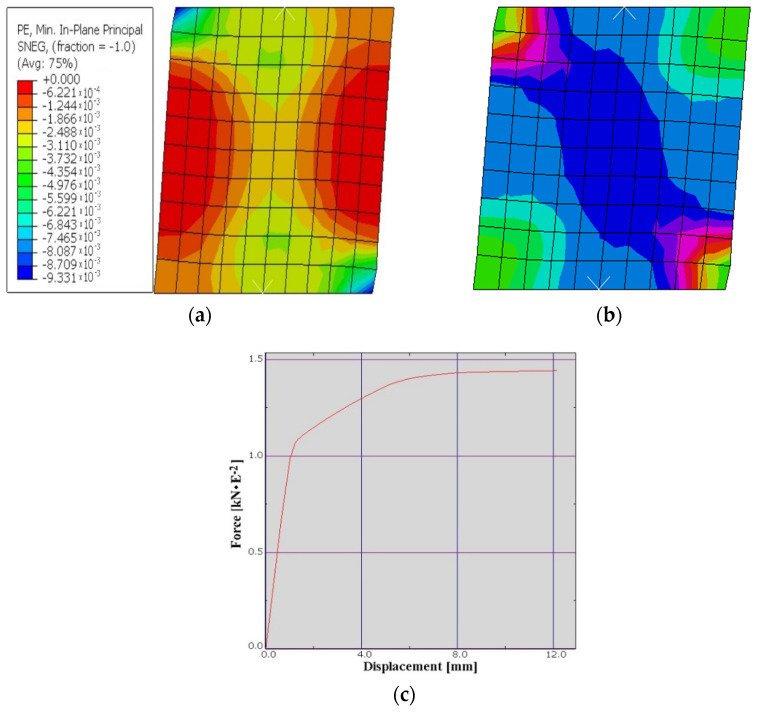
Compression plastic strain (**a**); strain components at integration point (**b**); graph of force–displacement to VHB (**c**).

For the wall realized of masonry of ceramic blocks with vertical holes of the Porotherm type, a numerical model was realized that should validate the experimental test carried out in the Civil Engineering and Installation Department from Timisoara. In the calculation, we used an average compressive strength of 10 [N/mm^2^] for the masonry wall from ceramic blocks with vertical holes. Moreover, in the analysis we used a plastic strain of 0.0025, a failure ratio R1 = 1.16; R2 = 1.08, and tension stiffening with displacement of 1.5. The modulus of elasticity of the ceramic block was of E = 16000N/mm^2^ with a Poisson ratio of 0.25. The numerical simulation was performed by applying a constant vertical force V = 150 kN and an increasing horizontal force until the failure. The results of the numerical analysis performed show that at the moment of wall failure the displacement at the top of the element was 12.17 mm. Making a comparison with the displacement of the element obtained from the experimental test, the ultimate displacement of which was 11 mm, it can be said that the results are almost identical which confirms veracity of the experimental test. At the same time the mode of failure is a classic one, with failure at shear force by generating a main stress level on the diagonal of the tested element. The results of the numerical analysis they are illustrated in the following images (Figure 4a,b) as well as in the graph in (Figure 4c).

### 4.3. Masonry Wall from Concrete Blocks with Wood Chips CBWC1

**Figure 5:**
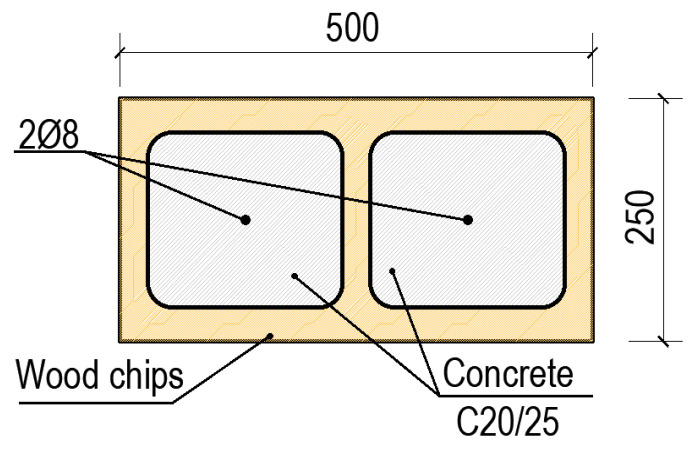
Configuration of block CBWC1.

In order to numerically evaluate the walls made of concrete blocks with wood chips, a numerical model was made than can be replicate the mechanical characteristics of concrete blocks with wood chips. Thus, the model realized has the following dimensions: length = 150 [cm]; height = 150 [cm]; thickness = 25 [cm], being made from blocks with dimension of 250 mm × 250 mm × 500 mm. After a few of simulations performed on the configuration of the concrete blocks with wood chips it was decided to adopt the solution from Figure 5, the solution in which the wood chips block is full with concrete class C20/25 and reinforced with two steel bars of diameter Φ8.

The concrete block with wood chips consists of a percentage of 41.80% wood chips and 58.20% of concrete. The block used without the addition of concrete has a compressive strength of 2.5 N/mm^2^, while the compressive strength of concrete is 20 N/mm^2^, and in the calculation using an average strength of 12.69 [N/mm^2^]. Moreover, in the analysis we used a plastic strain of 0.0035, a failure ratio R1 = 1.21; R2 = 1.08, and tension stiffening with displacement of 1.5. The modulus of elasticity obtained from the equivalence between the two materials was of E = 21,640 [N/mm^2^] with a Poisson ratio of 0.25. The numerical simulation was performed applying a constant vertical force V = 200 kN and an increasing horizontal force until failure.

**Figure 6:**
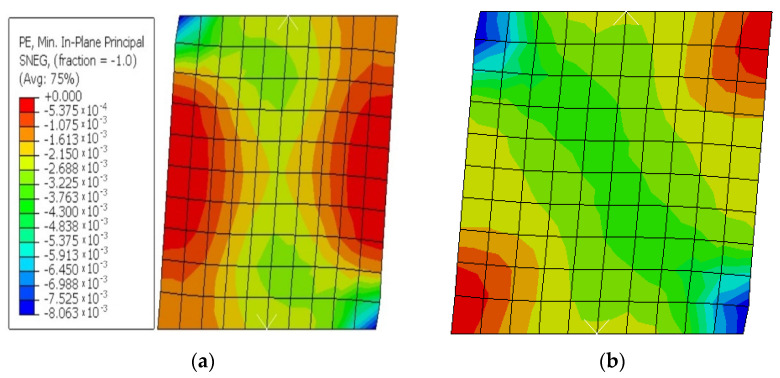

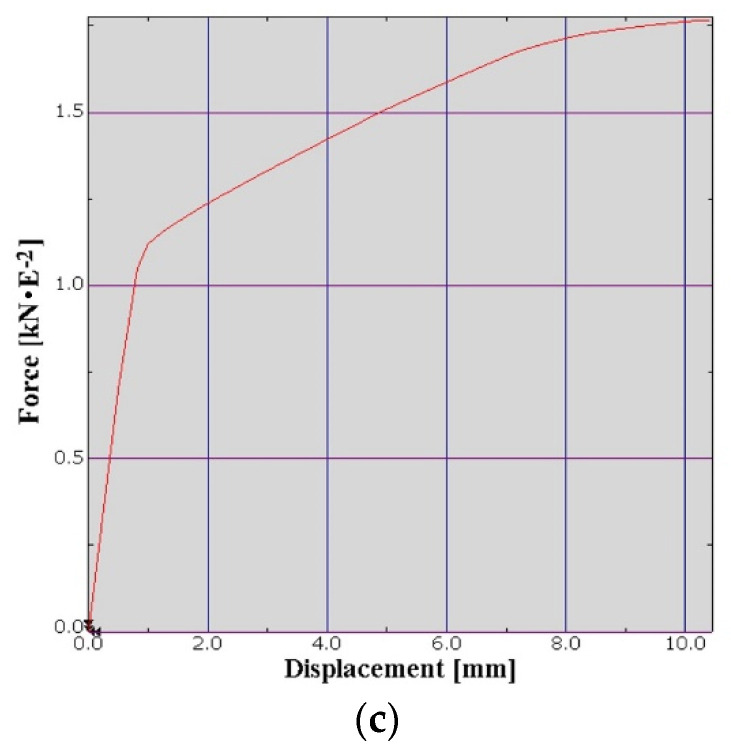
Compression plastic strain (**a**); strain components at integration point (**b**); graph of force–displacement to CBWC1 (**c**).

The results of the numerical analysis show that in the failure moment of the wall, the displacement at the top of the element was of 10.40 mm and the maximum horizontal force recorded was of H = 177.8 kN. In the following images there are illustrated the deformed element with the displacement at the top (Figure 6), the deformation mode as well as the area where the failure of the element is to occur (Figure 6b), as well as the force–displacement graph related to the numerical analysis performed (Figure 6c).

### 4.4. Masonry Wall from Concrete Blocks with Wood Chips CBWC2

**Figure 7:**
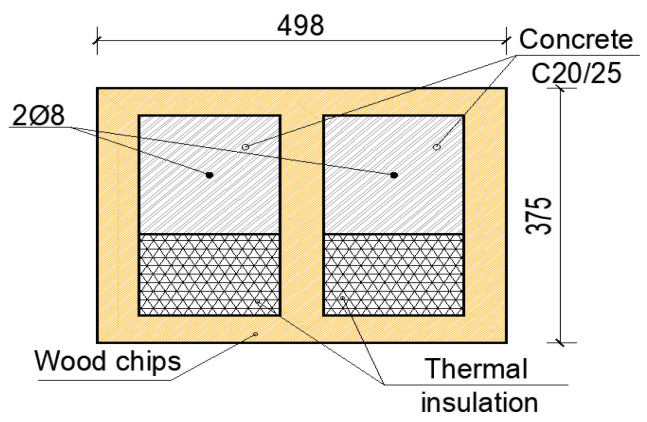
Configuration of block CBWC2.

The CBWC2 wall it consists of a block with wood chips with a different geometric configuration than the CBWC1 wall. Thus, the realized model has the following dimensions: length = height = 150 [cm]; thickness = 37.5 [cm], being made from blocks with dimension of 375 mm × 250 mm × 498 mm. After a few simulations performed on the configuration of the concrete blocks with wood chips it was decided to adopt the solution from Figure 7, the solution in which the block with wood chips it is partially filled with concrete, class C20/25, and reinforced with two steel bars of diameter Φ8 and thermal insulation.

**Figure 8:**
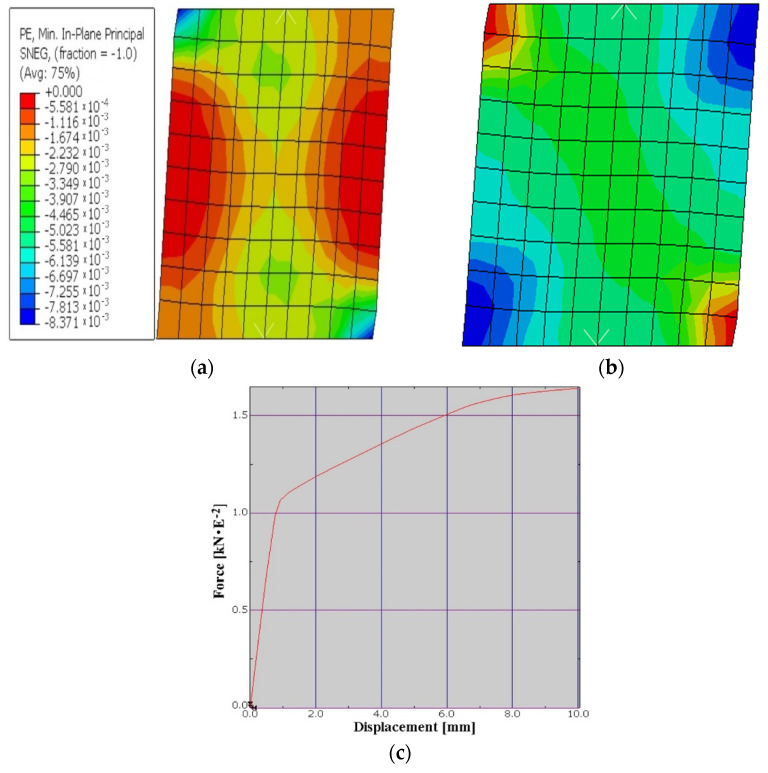
Compression plastic strain (**a**); strain components at integration point (**b**); graph of force–displacement to CBWC2 (**c**).

The concrete block with wood chips consists of a percentage of 45.98% wood chips, 32.05% of concrete, and 21.98% thermal insulation. In the calculation using an average compressive strength of 7.63 [N/mm^2^]. Moreover, in the analysis we used a plastic strain of 0.0035, a failure ratio R1 = 1.16; R2 = 1.08, and tension stiffening with displacement of 1.5. The modulus of elasticity obtained from the equivalence between the materials was of E = 14,215 N/mm^2^ with a Poisson ratio of 0.25. The test procedure was the same as that of the CBWC1 wall. The results of the numerical analysis show that in the failure moment of the wall, the displacement at the top of the element was of 9.99 mm, and the maximum horizontal force recorded was of H = 165.2 kN. In the following images there are illustrated the deformed element with the displacement at the top (Figure 8), the deformation mode as well as the area where the failure of the element is to occur (Figure 8b), as well as the force–displacement graph related to the numerical analysis performed (Figure 8c).

**Figure 9:**
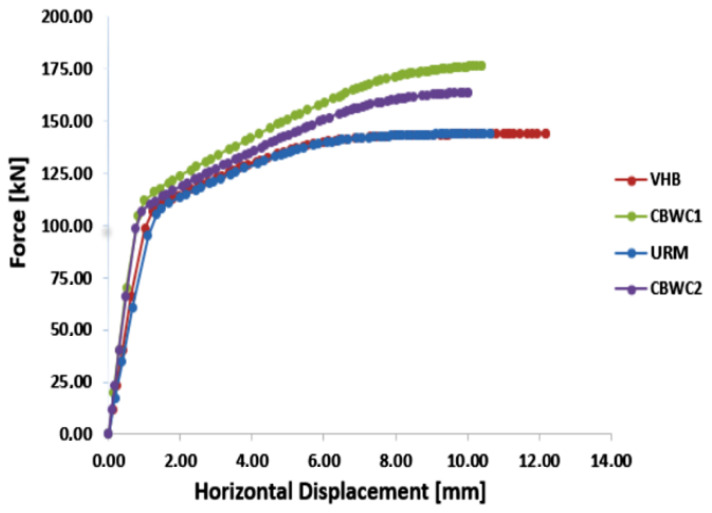
Graph of force–displacement for all four types of masonry blocks.

After obtaining the numerical results for the four types of blocks, a graph such as the one in Figure 9, was made to be able to make a comparison between solutions to highlight their performance from a structural point of view.

## 5. Conclusions

Following the theoretical study carried out on the walls made of concrete blocks with wood chips a series of aspects were observed that will be detailed in the following. Thus, performing the numerical analysis on the walls made up of the four types of masonry blocks the conclusions are as follows:

- The wall made of solid brick masonry and the wall made of bricks with vertical holes have a similar behavior, practically fail at almost the same horizontal forces, with the ultimate horizontal displacement being higher in the case of wall made of masonry brick with vertical holes.
- The solution of integral filling with concrete class C20/25, blocks with wood chips, obtained the best behavior between those four types of blocks. According to Figure 9, the wall made of blocks with wood chips integral filling with concrete, failed at a horizontal load of 177.8 kN, while the ultimate horizontal force recorded for the other three types of walls was between 144 kN and 165.2 kN.
- The force at which elements made of concrete blocks with wood chips failed was 23.5% higher than the force at which elements made of traditional brick failed; moreover, the ultimate horizontal displacement for elements made of concrete blocks with wood chips was 6.5% lower than for elements made of traditional brick.
- The solution of CBWC2 is previewed to be a beneficial solution, both mechanically and energetically, with this solution being in trend with the latest European directives on sustainability and energy building efficiency.

In conclusion, it is proposed to carry out an experimental program and to perform experimental tests on walls such as CBWC1 and CBWC2, made of blocks with wood chips filling with concrete class C20/25 and thermal insulation, for the validation of the results from the numerical analysis. We plan to perform additional experimental test on walls completely built with concrete blocks with wood chips in order to gain a better understanding of their physical characteristics under a seismic load. This will allow us to draw a wider range of conclusions on the behavior of concrete blocks with woodchips used in seismic areas.

## Figures and Tables

**Table 1:** Composition of concrete block with wood chips.

Type of Materials	Ratio
Wood chips	85%
Cement	10%
Polymer	5%

**Table 2:** Mix ratio of concrete use.

Type of Materials	Ratio
Cement	10%
Water	18%
Sand	25%
Gravel	41%
Air	6%

## Data Availability

The data that support the findings of this study are available from the corresponding author upon reasonable request.
